# Epidemiology of invasive *Haemophilus influenzae* serotype a disease in the North American *Arctic*, 2006–2017

**DOI:** 10.1080/22423982.2022.2150382

**Published:** 2022-12-02

**Authors:** Tammy Zulz, Grace Huang, Karen Rudolph, Carolynn DeByle, Raymond Tsang, Shalini Desai, Stephanie Massey, Michael G Bruce

**Affiliations:** a Arctic Investigations Program, Division of Preparedness and Emerging Infections, National Center for Emerging and Zoonotic Infectious Diseases, Centers for Disease Control and Prevention, Anchorage, Alaska, USA; b Infectious Disease Programs Branch, Public Health Agency of Canada, Ottawa, ON, Canada; c National Microbiology Laboratory, Winnipeg, MB, Canada; d Section of Epidemiology, Division of Public Health, Alaska Department of Health & Social Services, Anchorage, Alaska, USA

**Keywords:** Haemophilus influenzae, Hia, Indigenous, Alaska, Canada, incidence, Arctic

## Abstract

Invasive *Haemophilus influenzae* type a (iHia) disease was detected in Alaska and Northern Canada in 2002 and 2000, respectively. From 2006 to 2017, 164 iHia cases (Alaska=53, Northern Canada=111) were reported. Rates of iHia disease per 100,000 persons were higher in Northern Canada compared to Alaska and were significantly higher in Indigenous (Alaska 2.8, Northern Canada 9.5) compared to non-Indigenous populations (Alaska 0.1, Northern Canada=0.4). Disease rates were highest in Indigenous children <2 years of age (Alaska 56.2, Northern Canada=144.1) and significantly higher than in non-Indigenous children <2 (Alaska 0.1, Northern Canada 0.4). The most common clinical presentation in children <5 years was meningitis of age and pneumonia in persons ≥5 years old. Most patients were hospitalised (Alaska=87%, Northern Canada=89%) and fatality was similar (Alaska=11%, Northern Canada=10%). MLST testing showed sequence types ST23 and ST576 in Northern Canada and ST576, ST23 and ST56 in Alaska. Alaska and Northern Canada have high rates of iHia disease. A vaccine is needed in these regions to protect young children.

## Introduction

*Haemophilus influenzae* is a bacterial pathogen that can cause a range of illnesses from respiratory infections to severe invasive diseases such as meningitis, pneumonia and septic arthritis [1]. The organism can be encapsulated or non-encapsulated (non-typeable); the encapsulated strains are labelled as serotypes a–f, with serotype b (Hib) historically identified as the leading cause of bacterial meningitis in children in the USA and Canada [2]. Indigenous people in Alaska and Northern Canada have an increased risk for Hib disease and, prior to the introduction of the Hib vaccine, had some of the highest rates of Hib disease in the world [3–5]. Incidence of invasive disease caused by this pathogen has declined dramatically since vaccine introduction, however, increases in disease caused by other serotypes, in particular, serotype a (Hia), have been noted [6,7]. Conjugate vaccines for Hib do not provide protection for Hia [8]. Collaborative data sharing through the International Circumpolar Surveillance (ICS) project has highlighted the emergence of this serotype in Indigenous populations in Alaska and Northern Canada [9,10]. Here, we report on the continuing occurrence of invasive Hia disease in Arctic populations.

## Methods

ICS is an international network of Arctic regions that share public health surveillance data on invasive bacterial diseases including *Streptococcus pneumoniae, Haemophilus influenzae, Neisseria meningitidis*, and groups A and B streptococci [11–13]. Participants include northern provinces and territories in Canada (northern Labrador, northern Québec, Northwest Territories, Nunavut, and Yukon), Finland, Greenland, Iceland, Norway, Sweden (Norrbotten and Vasterbotten) and the US Arctic (Alaska). Network members collect cases through their respective surveillance systems and submit data to ICS headquarters at the US Centers for Disease Control and Prevention’s Arctic Investigations Program in Anchorage, Alaska, for compilation, analysis, reporting and publication. The ICS network was established in 1999; collection of *H. influenzae* data began in 2000. Participants in Canada, Greenland, Finland, Norway, Sweden and the US Arctic submitted data on *H. influenzae*. This analysis used only data from Canada and the US Arctic; the other participating regions either had no or very few Hia cases (Greenland, n=0, Norway, n=5) or did not provide serotype data (Finland, Sweden). A previous publication described Hia in the North American Arctic during the years 2000–2005 [9]; this manuscript reports on cases identified during the years 2006–2017.

Annualised incidence rates were calculated using population estimates for Northern Canada and Alaska obtained from Statistics Canada [14], and the Alaska Department of Labor and Workforce Development [15], respectively. The Indigenous population denominator for Northern Canada was estimated using the Census of Population estimates and the 2011 National Household Survey from Statistics Canada [14]. The estimated populations for Northern Canada and Alaska were 154,823 and 714,394, respectively; Indigenous people comprised 60% of the population in Northern Canada and 19% in Alaska.

A case of invasive *H. influenzae* disease was defined as illness in a resident of the surveillance area from whom *H. influenzae* was isolated or identified by polymerase chain reaction (PCR) from a normally sterile site sample including blood, cerebrospinal fluid, pleural fluid, peritoneal fluid, or joint fluid. Clinical manifestations of *H. influenzae* were determined by a review of the patient’s medical record.

Isolates from cases of invasive *H. influenzae* were forwarded to reference laboratories in Alaska and Northern Canada for species confirmation, serotyping and multilocus sequence typing (MLST). In Alaska, *H. influenzae* was confirmed by using Gram stain and factor X and V requirements (Differentiation Disks; Difco Laboratories, Detroit, MI, USA); a similar method is used in Canada (Oxoid, ON, Canada). Capsular serotyping was performed by slide agglutination with Difco antisera (Difco, Detroit, MI, USA) in ALASKA and (Difco, Oakville, ON, Canada) in Northern Canada (except the Laboratoire de Sante Publique in Quebec which used PCR). Serotype results in Alaska and Northern Canada were confirmed by PCR; PCR amplification of serotype-specific genes was done by using primers and probes as reported by Maaroufi, et al. [16] (Alaska) or Falla, et al. [17] (Northern Canada). MLST of seven housekeeping gene loci was done on all invasive Hia isolates in Alaska and Northern Canada according to a previously described method [18]. The sequence type (ST) assignments were made by using the *H. influenzae* MLST website (http://haemophilus.mlst.net/).

## Results

### Overall Hi

**Figure 1.**
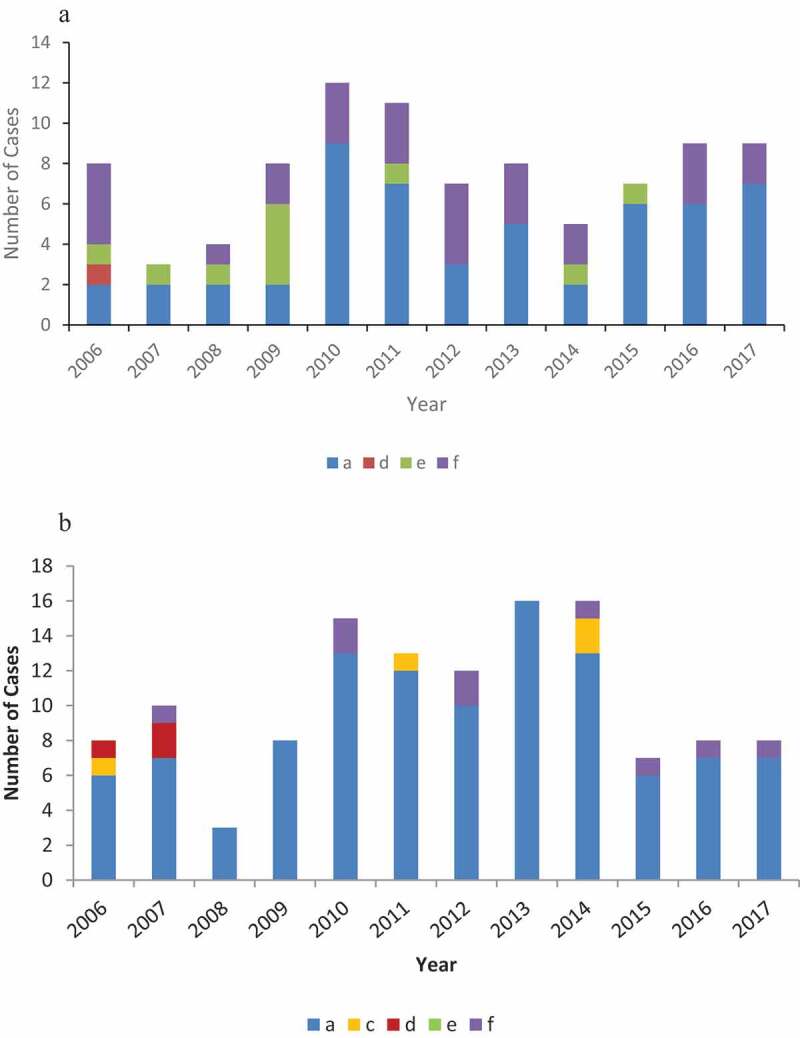
(a) Reported cases of non-b encapsulated invasive Hi disease by serotype, Alaska, 2006–2017. (b) Reported cases of non-b encapsulated invasive Hi disease by serotype, Northern Canada, 2006–2017.

A total of 430 cases (Alaska n=253, Northern Canada n=177) of invasive Hi were identified during 2006–2017; 412 isolates from these cases (Alaska n=241, Northern Canada n=171) were available for serotyping. Of these, 151 isolates (37%) were non-typeable (Alaska n=127, Northern Canada n=24); the remaining 261 isolates were 164 (63%) Hia (Alaska n=53, Northern Canada n=111); 43 (17%) Hib (Alaska n=23, Northern Canada n=20), 4 (2%) Hic (Alaska n=0, Northern Canada n=4), 4 (2%) Hid (Alaska n=1, Northern Canada n=3), 10 (4%) Hie (Alaska n=10, Northern Canada n=0) and 36 (14%) Hif (Alaska n=27, Northern Canada n=9) (Figure 1(a,b)).

### Descriptive epidemiology

Among the 53 Alaska invasive Hia cases, 44 (83%) occurred in children <2 years of age, 5 (9%) occurred in children 2–4 years of age and the remaining 4 cases occurred in persons ranging from 8 to 77 years of age (Table 1). Median age was 0.9 years (10.8 months), and 62% of cases were male. In children <5 years old, 94% (n=45) were age appropriately vaccinated for Hib. Forty-eight (91%) cases occurred in Indigenous people. Six cases (11%) were fatal; three occurred in children <1 year old, two in children 2–4 years of age and one was an adult. In Northern Canada, 66% (n=73) of the 111 invasive Hia cases occurred in children <2 years of age, 9 (8%) occurred in children 2–4 years of age and the remaining 29 (26%) cases occurred in persons ranging from 5 to 80 years of age (Table 2). Median age was 1.4 years, and 52% of cases were male. In children <5 years old, 95% (n=69) were age appropriately vaccinated for Hib; 96% (n=107) of cases occurred in Indigenous people. Ten (10%) of the 100 cases with known outcome data were fatal; five cases occurred in children <1 year of age, one case was 2–4 years of age and four in persons >5 years old (Table 1).

**Table 1.** Characteristics of Alaskan and Northern Canadian persons with invasive Hia disease, 2006–2017.

Characteristic	Alaska (n=53)	N. Canada (n=111)
Median age in years (min-max)	0.9 (0.1–76.8)	1.4 (0.1–80)
Sex, male, # (%)	33 (62)	58 (52)
^1^Indigenous, # (%)	48 (91)	107 (96)
^2^Age appropriately vax for Hib, # (% vax)	45 (94)	69 (95)
^3^Hospitalized, # (%)	46 (87)	94 (90)
^4^Deaths, # (%)	6 (11)	10 (10)

^1^Ethnicity missing/unknown for 1 case in N. Canada

^2^Vaccine history missing from 1 case in Alaska; denominator=48

Vaccine history missing from 9 cases in N. Canada; denominator=73

^3^Hospitalization data missing for 5 cases in N. Canada

^4^Death data missing/unknown for 11 cases in N. Canada

**Table 2.** Cases and annualised incidence rate of invasive Hia disease by age and ethnicity, 2006–2017.

Demographic group	Alaska, # cases (rate)^1^	N. Canada, # cases (rate)^1^
Overall	53 (0.6)	111 (6.0)
<2 years	44 (16.9)	73 (109.8)
<5 years	49 (7.5)	82 (50.1)
Overall, Indigenous	48 (2.8)	107 (9.5)
<2 years, Indigenous	42 (56.2)	72 (144.1)
<5 years, Indigenous	45 (24.4)	81 (64.8)
Overall, non-Indigenous	5 (0.1)	3 (0.4)
<2 years, non-Indigenous	2 (1.1)	1 (6.1)
<5 years, non-Indigenous	4 (0.9)	1 (2.6)
Overall, Indigenous v non-Indigenous	p<0.001	p<0.001
<2 years, Indigenous v non-Indigenous	p<0.001	p<0.001
<5 years, Indigenous v non-Indigenous	p<0.001	p<0.001

^1^Cases per 100,000 population

### Incidence rates

In Alaska, the overall annualised crude invasive Hia incidence rate was 0.6 cases per 100,000 population (Table 2). In Alaska children less than 5 years old, the rate was 7.5 per 100,000 population and in children less than 2 years old, the rate was 16.9 per 100,000 population. In Indigenous children less than 2 years old, the rate was 51 times higher than that in non-Indigenous children (p<0.001). The overall annualised crude invasive Hia incidence rate in Northern Canada was six cases per 100,000 population. In Northern Canada children less than 5 years old, the rate was 50.1 per 100,000 population and in children less than 2 years old, the rate was 109.8 cases per 100,000 population. There was one case in Northern Canada non-Indigenous children less than 2 years old (6.1 per 100,000) compared to 73 cases (144.1 cases per 100,000) in Indigenous children in this age category. When stratified by race, rates were consistently significantly higher in the Alaska and Northern Canada Indigenous population than in non-Indigenous population.

### Clinical illness

In children under the age of 5 years, the most common clinical illnesses due to invasive Hia infection were meningitis (45% Alaska, 27% Northern Canada), bacteraemia (26% Northern Canada) and pneumonia (25% Alaska). In individuals over the age of 5 years, pneumonia (25% Alaska, 46% Northern Canada) and bacteraemia (50% Alaska, 28% Northern Canada) were the most common clinical illnesses (Table 3). Eighty-seven per cent of all cases were hospitalised in Alaska and 90% in Northern Canada (Table 1).

**Table 3.** Clinical illnesses^1^ in Alaska and Northern Canada persons with invasive Hia by age, 2006–2017.

Illness	Alaska		N. Canada^2^	
	<5 years (n=49), # (%)	≥5 years (n=4), # (%)	<5 years (n=82), # (%)	≥5 years (n=29), # (%)
Meningitis	22 (45)	0 (0)	22 (27)	2 (7)
Pneumonia	12 (25)	1 (25)	20 (24)	13 (46)
Cellulitis	8 (16)	1 (25)	3 (4)	3 (11)
Septic arthritis	8 (16)	0 (0)	14 (17)	3 (11)
Bacteraemia	4 (8)	2 (50)	21 (26)	8 (28)
Osteomyelitis	2 (4)	0 (0)	1 (1)	0 (0)
Other^3^	2 (4)	0 (0)	2 (2)	1 (4)

^1^Cases may have multiple illnesses associated with Hia infection.

^2^10 Northern Canada cases are missing clinical diagnosis data.

^3^Other includes Empyema, unknown and “Other”.

### Subtyping data

**Figure 2.**
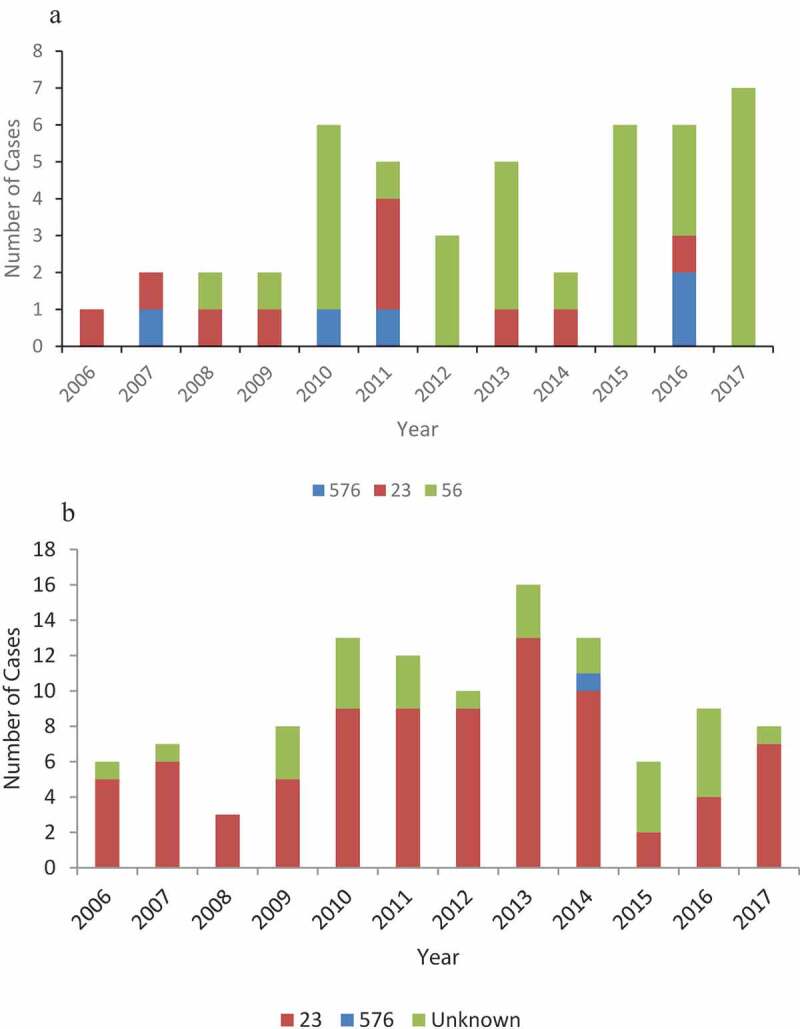
(a) Sequence type results for invasive Hia strains, Alaska, 2006–2017. (b) Sequence type results for invasive Hia strains, Northern Canada, 2006–2017.

Subtyping of the Hia isolates by MLST showed that the Alaska isolates were comprised of three STs: ST576 (n=5, 11%), ST23 (n=10, 21%) and ST56 (n=32, 68%) (Figure 2a) and of the 83 Northern Canada isolates with MLST data, 82 isolates were sequence type ST23 and 1 isolate was sequence type ST576 (Figure 2b).

## Discussion

Hia was described in the literature as early as 1931 [19], however, increases in incidence were not noted until the post-Hib vaccine era [7,9,10,20,21]. Cases of invasive Hia have been detected worldwide, and the severity of disease caused by this infection, similar to Hib, warrants consideration of a vaccine for control [21–25]. In the USA alone, the incidence of invasive Hia disease has increased more rapidly since 2002 (13% annually) than disease caused by other non-b serotypes or nontypeable strains [20,26]. Hib vaccine uptake among Hia cases in Alaska and Northern Canada suggests that if an Hia vaccine is developed, a high level of coverage could be achieved.

While secondary transmission of Hib was well known [27,28], epidemiologically linked Hia cases have only recently been reported in the literature [26,29]. Although there are no formal guidelines for control measures around Hia cases, the American Academy of Pediatrics Committee on Infectious Diseases has recommended that clinicians consider chemoprophylaxis for household contacts of a person with invasive Hia disease, as is recommended for Hib cases [30]. The State of Alaska Department of Health and Social Services, Division of Public Health, has endorsed these recommendations for household contacts of confirmed invasive Hia cases in Alaska following an outbreak of Hia in a remote Alaska village [31]. In Northern Canada, recommendations differ by region; in the Northwest Territories, Nunavik and Yukon, prophylaxis is recommended only for contacts of Hib cases; in Labrador and Nunavut, prophylaxis is recommended for contacts of any invasive Hi case prior to serotype results due to the lag time between case identification and lab testing (personal communication).

Our data show that invasive Hia continues to occur and be a concern in Arctic populations, particularly in Indigenous peoples. When compared to rates published in our previous collaborative analysis [9], overall incidence in Alaska and Northern Canada has increased significantly; in Alaska the overall rate increased from 0.3 cases per 100,000 (2000–2005) to 0.6 cases per 100,000 (2006–2017) (p<0.001) and in Northern Canada the overall rate increased from 3.9 cases per 100,000 (2000–2005) to 6 cases per 100,000 (2006–2017) (p=0.04). Data from this analysis are consistent with previous collaborative work showing a high relatedness among strains [9]; our current data demonstrate that 56 is the predominant sequence type in Alaska and 23 in Northern Canada; these two sequence types are known to be highly related [21]. Indigenous peoples are over-represented in the occurrence of invasive Hia in Alaska and Northern Canada, comprising 91% and 96% of reported cases, respectively. The higher overall rates of invasive Hia in Northern Canada may be attributed to the higher proportion of Indigenous people (60%) in the population than in Alaska (19%). Hia incidence rates in Indigenous peoples are significantly higher overall, particularly in children, than in non-Indigenous persons. Reports published since the late 1990s have shown that Hia disproportionately affects Indigenous populations in North America and Australia [5,7,20,32].

The severity of disease caused by Hia invasive infection is comparable to that seen in invasive Hib cases prior to the introduction of the Hib vaccine. Meningitis is the most common disease in children less than 5 years old with Hia infection in Alaska and Northern Canada, presenting in 45% and 27% of cases, respectively. Studies describing the epidemiology of invasive Hib disease in Alaska and Northern Canada pre-vaccine also report meningitis as the most common disease associated with the bacterial infection as well as case fatality rates that are similar to invasive Hia infections [33,34]. Virulence associated with Hia infections has been previously linked to the bexA deletion [35,36]; however, this genetic mutation has not been found in previously tested Hia isolates from Alaska and Northern Canada [9,10]. Increased risk of respiratory infections in populations that experience household crowding, lack of in-home piped water, poor indoor air quality, remoteness, and poverty may contribute to increased rates and severity of Hia disease [5,23,37,38].

This study is limited by the lack of historical data available for non-b invasive *H. influenzae* disease prior to the introduction of Hib vaccine in Alaska and Northern Canada. Serotyping efforts at that time were primarily focused on identifying Hib cases; the non-b serotypes were often not specifically detected. This analysis does not include underlying condition data that might be helpful in identifying risk factors for invasive Hia disease. Although underlying condition data was collected during the time of this study, the data was not collected consistently.

## Conclusions

This paper is an update to a previously published collaborative analysis of invasive Hia disease in Alaska and Northern Canada. Our data show that invasive Hia continues to cause morbidity and mortality among peoples of the North American Arctic, particularly Indigenous children. The severity of this bacterial infection as well as its increasing prevalence highlights the need for an Hia vaccine for use in Northern Canadian and Alaskan children.
